# Evaluation of Orthorexia Nervosa and Symptomatology Associated with Eating Disorders among European University Students: A Multicentre Cross-Sectional Study

**DOI:** 10.3390/nu12123716

**Published:** 2020-12-01

**Authors:** Anna Brytek-Matera, María Dolores Onieva-Zafra, María Laura Parra-Fernández, Anna Staniszewska, Justyna Modrzejewska, Elia Fernández-Martínez

**Affiliations:** 1 Institute of Psychology, University of Wroclaw, 50-527 Wroclaw, Poland; 2 Department of Nursing, Physiotherapy and Occupational Therapy, Faculty of Nursing of Ciudad Real, Universidad de Castilla-La Mancha, 13071 Ciudad Real, Spain; mariadolores.onieva@uclm.es (M.D.O.-Z.); marialaura.parra@uclm.es (M.L.P.-F.); 3 Department of Experimental and Clinical Pharmacology, Medical University of Warsaw, 02-091 Warsaw, Poland; astaniszewska@wum.edu.pl; 4 Institute of Pedagogy, Faculty of Humanities and Social Sciences, University of Bielsko-Biała, 43-310 Bielsko-Biała, Poland; justyna.modrzejewska@wp.pl; 5 Department of Nursing, University of Huelva, 21071 Huelva, Spain; elia.fernandez@denf.uhu.es

**Keywords:** orthorexia nervosa, eating disorder pathology, cross-cultural study, university students

## Abstract

The objectives of the present study were to (1) evaluate prevalence of orthorexia nervosa (ON) in university students in Spain and Poland, (2) assess differences in ON and eating disorder (ED) pathology in both samples and (3) examine the relationship between ON and ED symptoms among Spanish and Polish university students. Eight hundred and sixty university students participated in the present study (*M*_age_ = 21.17 ± 3.38; *M*_BMI_ = 22.57 ± 3.76). The Spanish and Polish samples comprised 485 and 375 students, respectively. The Düsseldorf Orthorexia Scale and the Eating Disorder Inventory were used in the present study. ON prevalence rates of 2.3% and 2.9%, respectively, are found in the Spanish and Polish samples. Compared to Polish students, Spanish university students reported increased drive for thinness and lower body dissatisfaction, lower level of ineffectiveness and lower level of interpersonal distrust. ON was positively related to drive for thinness, bulimia, body dissatisfaction, perfectionism interoceptive awareness (in both Spanish and Polish students) and ineffectiveness (in Spanish students). Our findings suggest that ON significantly overlaps with ED symptoms, which is in line with recent studies. Longitudinal studies are needed to assess how ON develops in a sample of young adults and whether it develops in isolation of or in parallel with ED pathology.

## 1. Introduction

Eating behaviour is strongly influenced by social context [1]. Over the last several years societal attitudes towards healthy eating and dietary behaviour are changing, with an increasing emphasis on high-quality foods or ‘clean’ eating [2]. “Clean eating” (restrictive eating patterns focusing on the consumption of healthy, “pure” foods) may reflect vulnerability to a pathological fixation with healthy eating [3]. Orthorexia nervosa (ON) [4,5] is characterised by an obsessive focus on “healthy” or “pure” eating, strict avoidance of foods considered to be impure, unhealthy or improper, intense preoccupation regarding dietary practices, and very rigid dietary rules with violations causing exaggerated emotional distress [6]. Restrictive diet is not used for weight-loss purposes but is related to the pursuit of health. Preoccupation with either affirmative or restrictive dietary practices believed to promote health (which result in the exclusion of entire food groups), focus on food quality (not quantity), self-punishment when not complying with dietary rules, health complications (e.g., malnutrition), psychological disturbances (e.g., major depressive disorder [7]), and cognitive distortions [8] are features of ON [9].

Recently, Barrada and Roncero [10] described two differentiable dimensions in diet preoccupation, one associated with the pathological dimension of ON (OrNe) and the other connected to a nonpathological interest in healthy eating (healthy orthorexia; HeOr). OrNe evaluates the negative social and emotional impact of trying to achieve a rigid way of eating. This dimension represents a pathological preoccupation with a healthy diet, which leads to negative consequences such as self-punishment, social isolation, and guilt, whereas HeOr assesses the tendency to have a healthy, balanced diet and interest in doing so. HeOr represents a healthy interest with diet, which is independent of psychopathology (eating disorders, obsessive-compulsive disorder, and negative affect). In this context, ON may be considered as adaptive due to selective healthy eating and its social characteristics (orthorexic behaviours are relevant evolutionary strategies because they can enable earning recognition and honours from others [11]). Therefore, ON may be viewed (tentatively) as a healthy diet protecting one’s health but also as a phenomenon having negative influence on health [12], however, this viewpoint should be adopted with some caution, due to the limited number of available studies on these subjects, and should hence be further tested in the future studies.

The prevalence of ON has been evaluated with considerable inconsistency mainly due to the employment of the different tools for evaluating the same construct. Depending on the population being studied (e.g., university students, athletes, patients with eating disorders) and the instrument being used (ORTO-15) rates between 1% and 90% have been reported [13]. While the ORTO-15 [14] has, to date, been the most widely used questionnaire, the Düsseldorf Orthorexia Scale (DOS) [15] was recently implemented to assess the prevalence of ON. Assessments via DOS have found cross-sectional rates for ON between 1.5% [16] and 6.9% in the general population [17] and between 3.3% [18] and 10.5% [19,20] among university students (Table 1).

Although in the past two decades interest in ON has increased, at present there exists ongoing debate on whether to consider ON as a single syndrome, a variance of an eating disorder (ED)—an antecedent of anorexia nervosa (AN), a way to maintain AN or a consequence of AN—or obsessive-compulsive disorder [24,25,26], a new variant of eating behaviour [27] or merely a culturally influenced attitude rather than a disease [28]. Previous studies have found a behavioural and symptomatology overlap between ON and AN [10,29,30] and association between ON and disordered eating behaviours and attitudes toward food [6,31,32,33]. The major discrepancy between ON and AN occurs in the main goal: being healthy (preoccupation with consuming healthy and pure foods) versus weight loss (preoccupation with weight loss). In addition, ON is related to obsession about quality of food intake, whereas AN is associated with obsession about the quantity of food intake [34]. Food and eating preoccupation, dietary restrictions, making eating-related issues the primary focus of one’s own life, intense anxiety regarding certain foods and their avoidance, sense of superiority over others based on one’s own eating practices, need for control, obsessive-compulsive personality traits (e.g., perfectionism, rigidity) and ego-syntonic nature are common features for both ON and AN [35]. Moreover, neurocognitive deficits (impairments in set-shifting, external attention, working memory) [25,36], cognitive distortion (e.g., magical beliefs about food) as well as similar symptomatology are similar in ON and AN patients, which may indicate analogous brain dysfunction in these individuals [37].

Both eating habits (e.g., avoidance of certain type of foods as saturated fats or animal fat products, following a strict eating schedule, spending large amounts of time preparing meals) and disordered eating habits are related to greater ON [26]. Promoting a healthy diet and acquisition of healthy eating habits in young adults is an important challenge because of dietary pattern changes (breakfast skipping, eating outside home) and nonadherence of dietary guidelines (despite positive attitudes towards them) [38]. Since eating habits formed in young adulthood (study period) shape eating behaviours during later life [39] and eating attitudes and behaviours constitute a common health problem among university students, there is a need for more comprehensive studies to determine the nature of ON (and to identify ways of overcoming it). Nowadays there is limited evidence on the relationship between ON (measured by DOS) and ED symptoms among university students. Although the influence of cultural differences on has been discussed (see the recent review by Strahler [13]), the interaction between sociocultural context and ON has been poorly examined. Previous study has shown that higher nutrition knowledge and higher education levels are positively associated with the Mediterranean dietary pattern [40,41]. In addition, research on food choice has found that Southern European populations give greater importance to sociability and cooking and enjoying food with others [42]. Cross-cultural studies have suggested that populations that prioritize pleasure over health demonstrate healthier eating behaviours [42,43]. One might expect that the Mediterranean diet together with convivial and social value attributed to eating [40] may play an important role in the Spanish sample.

The present study aimed to (1) evaluate ON prevalence (as measured by the DOS) in university students in Spain and Poland, (2) assess differences in ED symptoms (measured with the EDI) in both samples and (3) examine the relationship between ON and ED symptoms among Spanish and Polish university students. Based on the recent study conducted among Italian, Polish and Spanish university students [40] (to our knowledge, the only study of its kind available in the literature), we hypothesize that:

**Hypothesis 1** **(H1).** *ON prevalence will be higher among Polish students than Spanish ones.*

**Hypothesis 2** **(H2).** *Symptoms related to ED will be higher among university students in Poland than those in Spain.*

**Hypothesis 3** **(H3).** *A relationship between ON and ED symptoms will exist in both Spanish and Polish students.*

## 2. Materials and Methods

### 2.1. Study Design

A multicentre cross-sectional study was performed to analyse the ON construct and ED symptoms in a sample of European university students from Spain and Poland. For this purpose, a convenience sample was recruited, based on a joint collaboration between both countries. Firstly, each of the authors proposed the research protocol to the students attending their university classes (different types of university degrees were involved, as detailed below, thus ensuring a wider representation of the university population). The participants were evaluated using a protocol that included informed consent, demographic and anamnestic data collection sheets, and self-administered questionnaires.

### 2.2. Sample

A descriptive cross-sectional study was carried out. In total, 485 students (>18 years old) from the University of Castilla-La Mancha and 375 students (>18 years old) from two Polish universities were invited to participate in the study. The participants were recruited via informative talks delivered during university lectures in different faculties (nursing, law, chemistry, computer science and education in Spain and psychology, pedagogy, sociology in Poland). Enrolment in the study was voluntary and participants were requested to complete the online-survey developed using the JotForm platform (Spain) or through Google Forms (Poland). For ethical reasons, we were unable to inquire about the causes that led them to refuse to participate. The participants received no financial incentive. Written consent was obtained from the participants and the ethics committee of the University Hospital of Castilla-La Mancha (Code C-153) who approved the study according to the ethical guidelines outlined in the Declaration of Helsinki in 2013.

### 2.3. Instruments

#### 2.3.1. Demographic Information

Demographic information was gathered, including participants’ self-reported sociodemographic characteristics such as age, gender, variables such as weight and height. The average body mass index (BMI) was calculated based on the self-reported weight and height of participants.

#### 2.3.2. Düsseldorf Orthorexia Scale (DOS)

The DOS [15] is a unidimensional measure for assessing and screening ON. It includes ten items. The answers are based on a four-point Likert scale where 1 = never, 2 = rarely, 3 = often and 4 = always. The score ranges from 10 to 40 points. The cut-off point is ≥30 points for labelling the presence of ON. A score between 25 and 29 identifies a risk of developing ON, while a total score of less than 25 demonstrates the absence of ON [15]. The internal consistency of the original scale was 0.83. This scale was validated in both Spanish and Polish and the psychometric properties were considered adequate. The internal consistency for the Spanish version of the DOS is 0.841 [19] and for the Polish version (DOS-PL) is 0.840 [23].

#### 2.3.3. Eating Disorder Inventory (EDI)

The EDI [44] was designed to evaluate different cognitive and behavioural features of AN and bulimia nervosa (BN). The EDI consists of 64 items divided into eight subscales: drive for thinness, bulimia, body dissatisfaction, ineffectiveness, perfectionism, interpersonal distrust, interoceptive awareness and maturity fears. Each item is assessed according to a six-point scale (from “always” to “never”) with a score of 0–3. All subscales can be added together for an overall score or each subscale can be used separately. Both Spanish [45] and Polish version of the EDI [46] shows a satisfactory internal consistency (Spanish version: α = 0.65 to 0.93; Polish version: α = 0.65 to 0.92).

### 2.4. Statistical Analysis

A bivariate descriptive analysis was carried out with the sociodemographic and anthropometric data using frequencies and percentages for the qualitative variables and using mean and standard deviation for the quantitative variables comparing the samples of Spanish and Polish university students. The chi-square linear trend test was used to compare the qualitative variables with the BMI categorized as an ordinal variable. For the comparison of quantitative variables the Student’s *t*-test was used for independent samples. A comparison of the scores of the eight dimensions of the EDI was carried out among the students of both countries also by means of this last statistical test. Finally, a Pearson’s correlation was made between the DOS scores and the EDI dimensions in the samples from both countries and globally. The appropriateness of conditions for the application of the statistical tests employed was verified in all cases. The established significance level was 0.05.

## 3. Results

### 3.1. Sociodemographic and Anthropometric Results

A total of 860 university students participated, of which 43.6% (375) were Polish and 56.4% (485) were Spanish, 65.1% (560) were women and 34.9% (300) were men. The mean age of the total participants was 21.17 ± 3.38 (Min. 18–Max. 35) and their mean BMI was 22.57 ± 3.76 kg/m^2^. When categorized according to WHO references (WHO, 2000), 8.7% (75) were underweight (≤18.5 kg/m^2^), 71.2% (612) had normal weight (18.5–24.99 kg/m^2^), 15.3% (132) were overweight (25–29.99 kg/m^2^) and 4.8% (41) were obese (≥30 kg/m^2^). The comparison of sociodemographic and anthropometric data between countries is shown in Table 2.

### 3.2. ON and Sociodemographic and Anthropometric Variables

The mean DOS score was 17.50 ± 5.181 for the Spanish population and 17,49 ± 5.244 for the Poland sample. Based on the DOS, 89.5% (770) of the participants did not suffer or have a risk of ON (DOS score < 25), 7.9% (68) had a risk of ON (DOS score 25–29) and 2.6% (22) exceeded the preliminary cut of score of ON (DOS score ≥ 30), of which 50% (11) were Spanish and another 50% (11) were Polish. Therefore, in the group of Spanish students there was 2.3% of ON compared to 2.9% in Polish students (*p* > 0.05).

When comparing the ON trend in the different groups according to the BMI, no statistically significant differences were found either. However, most of the participants with ON had a normal weight, representing 72.7% in Spanish and 68.3% in Polish students (*p* > 05).

Regarding the participants classified as not having ON (838), 8.1% (68) were classified as at risk for ON according to the DOS questionnaire, of which 60.3% (41) were Spanish and 39.7% (27) Polish. When comparing the sociodemographic and anthropometric characteristics in students with risk of ON and without the same, no significant differences were found. Table 3 analyses sociodemographic and anthropometric results among university students without ON and with those at risk of ON in each country.

When comparing the mean score of each dimension of the EDI between Spanish and Polish university students, a higher mean score was identified in the Spanish sample for drive for thinness (*p* < 0.01) with a lower mean score for the body dissatisfaction (*p* < 0.01), ineffectiveness (*p* < 0.01) and interpersonal distrust (*p* < 0.01) dimensions (Table 4).

### 3.3. Correlations Between ON and ED Symptoms

Upon analysing the group of participants, a correlation was identified between the DOS scores and several dimensions of the EDI: drive for thinness (r = 0.170; *p* < 0.01), bulimia (r = 0.170; *p* < 0.01), body dissatisfaction (r = 0.187; *p* < 0.01), ineffectiveness (r = 0.103; *p* < 0.01), perfectionism (r = 0.146; *p* < 0.01) and interoceptive awareness (r = 0.188, *p* < 0.01). However, no correlation was identified with the scores of the interpersonal distrust dimension, nor maturity fear (Table 5). When independently analysing these aspects in the sample of each country, in the Spanish population the same correlations were significant; however, in Poland, the same correlations were found except for the ineffectiveness dimension (r = 0.075; *p* > 0.05).

## 4. Discussion

The effect of cultural differences on ON has been debated in the literature [13]. In addition, the most recently proposed ON definition criteria have already taken cultural aspects into consideration [34]. The present study aimed at investigating ON prevalence among university students in Spain and Poland. Out of the whole sample, ON was prevalent in 2.6% (50% of Spanish students and 50% of Polish students), while 7.9% exhibited the risk of developing ON (60.3% of Spanish students and 39.7% of Polish students). Our results showed that Spanish university students had an ON prevalence rate of 2.3%, whereas the Polish sample had an ON prevalence rate of 2.9%. Since our results were not statistically significant (*p* > 0.05), our first hypothesis was not confirmed. Our results are in contrast to recent findings [40] that showed ON prevalence to be higher among Polish students (66.5%) compared with Spanish one (18.8%). It is worth pointing out that those researchers [40] used a different ON self-report questionnaire (ORTO-15) and for this reason it is difficult to compare those results with the findings of the present study. The prevalence of ON among both Spanish and Polish university students is lower than the values observed in previous studies that employed the DOS to investigate German students (3.3%) [18], Chinese students (7.8%) [22] and U.S. students (8%) [21]. Moreover, in our recent studies 10.5% of Spanish students were classified as having ON [19], while ON was observed in 6.6% of Polish students [23]. To our knowledge, a total of six studies (published in English) used the DOS among adults from the general population [6,17,47,48,49,50] with ON prevalence ranging from 2.3% [48] to 8.4% [50]. The prevalence of ON appears to be increasing, and more research is needed to elucidate the cultural and social aspects of disordered eating habits in order to provide culturally appropriate psychological treatment.

The second objective of our study was to compare ED pathology in both samples. Our findings demonstrated that Spanish students reported a greater concern with dieting, preoccupation with weight and entrenchment in an extreme pursuit of thinness than the Polish sample. Meanwhile students in Poland presented higher body dissatisfaction, higher feelings of general insecurity, worthlessness and the feeling of not being in control of their life (ineffectiveness), and a greater sense of alienation and a higher general reluctance to form close relationships (interpersonal distrust) compared to the Spanish sample (H2 was partially confirmed). From these results one may expect that compared to their contemporaries in Spain, students in Poland present a higher risk of developing ED pathology. According to the cultural dimensions [51], some Western countries, like Spain, are likely to be closer to non-Western countries than to other Western countries, like Poland. The Individualism/Collectivism Index classifies Spain as a collectivistic society while Poland as an individualistic culture [51,52]. Collectivist cultures focus on the individual’s behaviour for the whole group (cooperative tasks) whereas individualistic societies are characterized by an emphasis on what makes the individual distinct (competitive tasks) [53]. Our findings are in contrast to previous findings [53] from which it was argued that individuals from individualist countries showed a higher drive for thinness and less body dissatisfaction than those from collectivist societies. Nevertheless, our results concur with other studies [54] showing that the thin ideal (directly related to body dissatisfaction and ED symptomatology) is due to Western influence. Based on our results we may expect that collectivist values minimize the expression of ED pathology [54] in our Spanish sample, while abnormal eating patterns might be more influenced by individualism cultural attitudes in our Polish sample.

It is worth pointing out that we used an older version of the EDI [44], which can be considered a limitation of this study. We used the EDI [44] following the recommendation of clinical psychiatrists specialized in eating disorders who recommended the use of an older version of EDI as it was the most used version in the clinical context of the countries where are study took place. Likewise, when we prepared the study, version III of the EDI was not yet available. In addition, in Poland, EDI II and EDI III were not available at the time of our study.

The third objective was to evaluate the relationship between ON and ED symptoms among Spanish and Polish university students. ON was positively linked to cognitive and behavioural features of ED among both samples. Drive for thinness, bulimia, body dissatisfaction, perfectionism, interoceptive awareness (in both Spanish and Polish students) and ineffectiveness (in Spanish students) were found to be related to ON (H3 was confirmed). These results are in line with other studies showing that greater ON symptomatology is associated with greater disordered (pathological) eating among adults from the general population [20,31,32,55], such as body dissatisfaction [47], bulimia [48] or restraint eating, eating concern, weight concern, and shape concern [6]. ON significantly overlapped with ED pathology in both of our samples, suggesting that ON should be consider as disordered eating behaviour closely connected with ED.

## 5. Conclusions

Our findings show that 2.3% of students in Spain and 2.9% of students in Poland exceeded the preliminary cut of score of ON, while 8.6% of the Spanish students and 7.4% of the Polish sample were at risk of developing ON. Our findings suggest that ON significantly overlapped with ED symptoms, which is in line with recent studies. Healthy eating and healthy lifestyles are viewed as desirable within society. There is also suggestion that Western societies have become orthorexic societies [56], therefore it is difficult to recognize when healthy eating becomes obsessional and problematic [57]. Individual health behavioural patterns that develop during transition from adolescence to adulthood (18–25 years) often persist into later life, influencing individuals’ health, that of their partners and/or children [38]. Thus, longitudinal studies are needed to assess how ON develops in a sample of young adults and whether it develops in isolation of or parallel to ED pathology.

## Figures and Tables

**Table 1:** Studies assessing orthorexia nervosa (ON) using the Düsseldorf Orthorexia Scale (DOS) among a sample of university students.

Authors	Country	N	Gender	Age	Prevalence of ON (%) Based on DOS across All Sample	Prevalence of ON (%) Based on DOS across Gender
					a. Presence of ON (≥30) b. At Risk of ON (25–29) c. No Risk of ON (<25)	
Depa et al. [18]	Germany	446	70% female 30% male	21.7 ± 2.6	a. 3.3% b. 9.0% c. 87.7%	1. 2.8% female 3.7% male 2. 10.4% female 5.9% male 3. na
Chard et al. [21]	United States of America	384	69.5% female 30.5% male	19.64 ± 2.58	a. 8% b. 12.4% c. 79.5%	na
He et al. [22]	China	1075	52.7% female 47.3% male	20.11 ± 1.01	a. 7.8% b. 18.2% c.	a. 5.3% female 10.6% male b. 14.5% female 22.4% male
Parra-Fernández et al. [19,20]	Spain	492	56.9% female 43.1% male	19.97 ± 3.03	a. 10.5% b. na c. 89.4%	a. 6.5% female 4.1% male b. na c. 93.5% female 95.9% male
Brytek-Matera, [23]	Poland	412	77.2% female 22.8% male	24.62 ± 6.86	a. 6.6% b. 11.9% c. 81.5%	a. 6.3% female 7.4% male b. 12.6% female 9.6% male c. 79.3% female 83% male

**Table 2:** Sociodemographic and anthropometric results in Spanish and Polish students.

Variables		Spain (n = 485)			Poland (n = 375)			*p* Value
		n	%	M ± SD	n	%	M ± SD	
Sex	Male	210	43.3	-	90	24	-	0.000 ^a,^**
	Female	275	56.7	-	285	76	-	
Age (years)	-	-	-	19.76 ± 2.18	-	-	22.99 ± 3.77	0.000 ^b,^**
BMI(kg/m^2^)	-	-	-	22.43 ± 3.54	-	-	22.76 ± 4.03	0.197 ^b^
BMI ranges	≤18.5	47	9.7	-	28	7.5	-	0.077 ^c^
	18.5–24.99	349	72	-	263	70.1	-	
	25–29.99	69	14.2	-	63	16.8	-	
	≥30	20	4.1	-	21	5.6	-	

^a^ Chi-squared test, ^b^ Student’s *t*-test; ^c^ chi-square linear trend test; ** *p* < 0.01.

**Table 3:** Comparing sociodemographic and anthropometric results among students having no risk of ON and students at risk of developing ON according to the DOS.

Variables		Spain (n = 474)				Poland (n = 364)	
		Having no Risk of ON	At Risk of Developing ON	*p*-Value	Having No Risk of ON	At Risk of Developing ON	*p*-Value
Sex n (%)	Male	190 (43.9%)	17 (41.5%)	0.766 ^a^	81 (24%)	5 (18.5%)	0.516 ^a^
	Female	243 (56.2%)	24 (580.5%)		256 (76%)	22 (81.5%)	
Age (years) M ± SD		19.79 ± 2.22	19.59 ± 1.76	0.563 ^b^	23.07 ± 3.87	22.41 ± 2.97	0.384 ^b^
BMI (kg/m^2^) M ± SD		22.45 ± 3.61	22.12 ± 2.98	0.568 ^b^	22.76 ± 4.80	22.25 ± 3.49	0.530 ^b^
BMI ranges n (%)	<18.5	41 (9.5%)	5 (12.2%)	0.552 ^c^	25 (7.4%)	3 (11.1%)	0.305 ^c^
	18.5–24.99	312 (72.1%)	29 (70.7%)		236 (70%)	20 (74.1%)	
	25–29.99	61 (14.1%)	6 (14.6%)		57 (16.9%)	3 (11.1%)	
	≥30	19 (4.4%)	1 (2.4%)		19 (5.6%)	1 (3.7%)	

^a^ Chi-squared test, ^b^ Student’s *t*-test; ^c^ chi-square linear trend test.

**Table 4:** Cognitive and behavioural features of eating disorder (ED) among students from Spain and Poland.

Dimensions	Countries	M ± SD	*p*-Value
Drive for thinness	Spain	5.08 ± 5.14	0.003 *
	Poland	4.05 ± 4.75	
Bulimia	Spain	2.54 ± 3.05	0.845
	Poland	2.59 ± 437	
Body dissatisfaction	Spain	5.82 ± 6.05	0.000 *
	Poland	7.73 ± 6.86	
Ineffectiveness	Spain	2.93 ± 4.02	0.000 *
	Poland	6.03 ± 5.08	
Perfectionism	Spain	5.33 ± 3.77	0.544
	Poland	5.16 ± 4.20	
Interpersonal distrust	Spain	3.00 ± 3.39	0.000 *
	Poland	4.87 ± 4.34	
Interoceptive awareness	Spain	4.55 ± 4.54	0.209
	Poland	5.01 ± 5.67	
Maturity fear	Spain	6.86 ± 4.49	0.994
	Poland	6.85 ± 5.22	

* *p* < 0.05.

**Table 5:** Correlation between ON and ED symptoms in the total sample.

	1	2	3	4	5	6	7	8	9
1.ON		0.414 **	0.170 **	0.187 **	0.103 **	0.146 **	0.036	0.188 **	−0.014
2. Drive for thinness			0.475 **	0.583 **	0.327 **	0.286 **	0.058	0.464 **	0.181 **
3. Bulimia				0.373 **	0.389 **	0.365 **	0.114 **	0.607 **	0.195 **
4.Body dissatisfaction					0.463 **	0.106 **	0.211 **	0.347 **	0.206 **
5. Ineffectiveness						0.270 **	0.489 **	0.577 **	0.343 **
6. Perfectionism							0.119 **	0.438 **	0.160 **
7. Interpersonal distrust								0.328 **	0.179 **
8. Interoceptive awareness									0.354 **
9. Maturity fear									

** *p* < 0.01.
